# A study of rural upbringing and education on the intent of health professional students to work in rural settings

**DOI:** 10.5116/ijme.50f1.48cf

**Published:** 2013-01-28

**Authors:** Alcinda K. Trickett-Shockey, Christina S. Wilson, Laura R. Lander, Greg A. Barretto, Grazyna D. Szklarz, Gail C. VanVoorhis, Joseph J. Minardi

**Affiliations:** 1 Department of Periodontics, Division of Dental Hygiene, School of Dentistry, West Virginia University, Morgantown, West Virginia, USA; 2 Department of Behavioral Medicine and Psychiatry, School of Medicine, West Virginia University, Morgantown, West Virginia, USA; 3 Department of Pediatrics, School of Medicine, West Virginia University, Morgantown, West Virginia, USA; 4 Department of Basic Pharmaceutical Sciences, School of Pharmacy, West Virginia University, Morgantown, West Virginia, USA; 5 School of Nursing, West Virginia University, Morgantown, West Virginia, USA; 6 Department of Emergency Medicine, School of Medicine, West Virginia University, Morgantown, West Virginia, USA

**Keywords:** Rural health practice, student training sites, student upbringing

## Abstract

**Objectives:**

To explore whether student rural upbringing, rural training were associated with their intent to practice in a rural health care setting after graduation in the disciplines of Dentistry, Dental Hygiene, Medicine, Nursing, and Pharmacy at a large university Health Sciences Center in Appalachia.

**Methods:**

Graduating healthcare professional students were surveyed to determine student rural background and training competency as compared to location of intended practice after graduation (N= 145). Differences were identified using descriptive statistics within and between healthcare disciplines using Spearman’s rank correlation and Kruskal-Wallis tests, Univariate, Chi-square and Pearson analyses for emergent themes or trends.

**Results:**

Positive attitudes towards rural rotations and competency training were found. Twenty-one percent respondents graduated from high school in a rural setting (<2,500), while 4% reported their intent to practice in that setting. Conversely, 52% reported intent to practice in >50,000 population, while 12% grew up in this community size.

**Conclusions:**

Student attitudes towards learning rural competency are positive; however, this survey suggests rural upbringing and training may not be sufficient to encourage health care professionals to practice in rural settings. More investigation is needed to determine effective incentives, perceived advantages or disadvantages and deterrents to rural healthcare practice.

## Introduction

Ongoing problems such as lack of health care access and health professional shortages have plagued rural areas in the United States for more than a century despite considerable attention to the problem.^1^^,^^2^ Health professional workforce shortages exist because small communities struggle to attract, support and retain well trained clinicians. Nearly 20% of the American population lives in rural communities, though only 9% of physicians practice there.^3^ Rural health professional shortages apply not only to physicians, but also include nurses, nurse practitioners, physician assistants, dentists, dental hygienists, pharmacists as well as all other allied health professionals. Generally, health professional shortages worsen as the community size shrinks, becomes more isolated or poor.^4^In West Virginia, 50 of the 55 counties have a HPSA (Health Professional Shortage Area) designation for primary care and 40 of the 55 counties have a HPSA designation in dentistry.^5^ Rural upbringing and rural professional training are two factors that have been shown to highly correlate with the recruitment and retention of health care workers and physicians in rural settings.^6^^-^^8^In an effort to address the increasing need for health care providers in rural areas, the West Virginia Rural Health Education Partnership (WVRHEP) was established in 1972. The mission of this educational partnership is to recruit, educate, and retain quality health care professionals for rural communities to improve the health of the people of West Virginia. The WVRHEP rural education training consists of didactic training and completion of a rural rotation. The rural rotation is experiential, where students train in a variety of rural community settings. Duration of rotation varies by discipline.Recently, Shannon and colleagues investigated student’s experiences at WVRHEP sites and found that 72% of the students rated the quality of their rural rotations as “very good” to “excellent”, and reported a greater sense of social responsibility after completing a rural rotation. WVRHEP utilizes 332 training sites in West Virginia in rural clinics or school settings.^9^ Across the country and worldwide, health professional training programs such as WVRHEP have been shown to have a positive impact on increasing the number of health care workers and physicians practicing in rural areas.^2^^,^^7^ Previous studies have suggested rural upbringing is predictive of intent to practice in a rural location.^2^^,^^6^^,^^10^^-^^13^The vast majority of the rural upbringing follow-up studies have been conducted within a single discipline, most commonly medicine. In contrast, Daniels and colleagues administered a retrospective follow-up survey to 1300 multidisciplinary health practitioners trained at a southwestern medical school.^10^ Analyses utilizing a dichotomous rural (<50,000) versus urban (>50,000) comparison derived from US Census Bureau criteria for federal health care policy, reportedly showed a positive association between rural childhood upbringing and choice of rural professional practice.The present study was designed to assess whether the rural background of health care students who receive rural training was associated with intent to practice in a rural setting. For the purposes of this project, rural was conservatively defined according to the most stringent population criteria for Census Urban Areas which “represents territories outside of urban areas with population less than 2500” according to review of U.S. Census year 2000 data.^14^ A review of previous rural studies on this topic revealed that many different definitions of rural have been utilized, making it difficult to compare results. This research project was innovative in that it sampled students about to complete their professional degrees from five health disciplines to learn about their intent to seek work in rural communities. Our methodology used more stringent and precise measures of rural community size to analyze the relationship between the population size of the communities in which health professional students were raised and intent for future practice in rural settings.^15^

## Methods

### Study design

A survey, consisting of twenty-nine questions developed by the authors, was designed to evaluate the factors which influence an individuals’ decision to practice in a rural setting. To facilitate question design and scope, a literature review was completed prior to the question creation. Using this knowledge, the authors created the survey questions. A pilot survey using the survey questions was administered. This was completed to test question validity and readability. A small, comparable sample student population (9 students) who were not in the target population was used. From this pilot study, identified survey questions were then analyzed for psychometric properties and modified to improve question validity, clarity, and reliability. The modified final survey provided an increase in accuracy to answer the original hypothesis to improve the psychometric properties.The final survey was distributed to University Health Sciences Center professional students nearing completion of their degrees in a cross-sectional design to determine student upbringing as well as opinions about their rural rotation experiences and their preference of future practice sites. No positive or negative implications of any possible effect of population size on practice characteristics were made to the survey participants to remove any perceived or real bias.Prior to conducting any part of the pilot survey or the final survey, the entire study proposal was submitted for IRB approval and received exempt status from the University Institutional Review Board. This status is on file with West Virginia University Compliance Department.An invitation to participate in this project was distributed electronically to the targeted participants through the University Health Sciences Center intranet system. Students were able to access the survey via an electronic link. Weekly follow-up email reminders and verbal announcements by faculty were made to encourage volunteer participation. One month after the survey was made available to the survey participants the survey was closed and the results were gathered for statistical analysis. Respondents did not receive remuneration, but after completing the survey, all were invited to take part in a drawing for a $75 bookstore gift card as an incentive. All responses were held in strict confidence and student participation was voluntary.

### Participants

All health sciences center graduating members from the five schools were eligible to participate in this survey. All 443 graduating members of the 2010-2011 academic year enrolled in five health disciplines were invited to complete an online survey about their rural background, rural training, cultural knowledge, and population size of the location where they intended to practice. A total of 145 students responded with 143 completing the entire questionnaire (see Table 1 for Professional Student Respondent Demographics). Students invited to participate included both graduate and undergraduate students from five different health professional disciplines: 48 Dentistry, 21 Dental Hygiene, 112 Medicine students, 178 Nursing, 84 Pharmacy. The majority of students had been actively involved in or had completed their WVRHEP rural rotation curriculum and training while others had yet to do so. WVRHEP faculties from all five disciplines were consulted to determine how each professional school participates in the WVRHEP program. It should be noted that each health professional discipline has highly variable didactic and course work preparation as well as differing length of rural rotation training. Fourth or final year students were chosen because these students complete a rural rotation during their last year of training.

**Table 1:** West Virginia University (WVU) health professional student respondent demographics (N = 145)

Student respondent demographics	N	%
Gender		
Male	44	30
Female	101	70
Age		
< 22 years	20	14
23 – 25 years	71	49
26 to 29 years	39	27
30 – 34 years	8	6
– 40 years	3	2
> 40 years	2	1
No response	2	1
Race/Ethnic Status		
White/Caucasian	126	87
Racial/Ethnic minority group member	17	12
No response	2	1
School/Program Attending		
Dental Hygiene	10	7
Dentistry	8	6
Medicine	49	34
Nursing	39	27
Pharmacy	37	26
No response	2	1
Size of Home town/City (High school)		
Population > 100,000	7	5
Population 50,000 to 100,000	10	7
Population 20,000 to 49,999	22	15
Population 10,000 to 19,999	38	26
Population 2,500 to 9,999	22	15
Population < 2,500	30	21
No response	16	11
Rural Rotation		
Yes	113	78
No	32	22

### Instrument

Portions of the survey were adapted from the SERFE (Student Evaluation of Rural Field Experience) used by WVRHEP to assess students’ rural rotation experience.^9^ Other survey items were influenced by previous rural health professional surveys, polling faculty from each professional school, as well as, pilot results from the nine health professional students.^16^ The primary focus of the survey was to collect information regarding rural cultural upbringing, rural training, and location of intent to practice among professional students. Respondents were informed that for the purpose of this survey, rural was defined as an area with a population of <2,500 residents based on the most stringent criteria for Census Urban Areas on the first page of the survey.^17^ Rural cultural competency and knowledge questions were included in the original research survey based on the content of the training materials presented to students in each discipline. It was determined though that those questions were not validated and would not be included in the present results.The instrument consisted of 29 questions. The first section consisted of 6 multiple choice and open-ended questions which gathered demographic data about the town in which they attended high school and the size of the setting in which they intended to practice their profession after completing their degree. The demographic data allowed us to identify differences between health professional disciplines as well as correlations between the population size of the community in which they grew up and their intent to practice in a rural setting. Included in the analyses are trends across professional disciplines. Reported town and state of high school graduation was used as a proxy for residential upbringing. High school town population was categorized by the investigators according to US Census 2000 data.^14^The second section of the survey contained 9 questions about culture competency, which were included in the statistical analysis, and 12 questions about cultural knowledge, which were not included in the statistical analysis. The questions were multiple choice styled with most of them being formatted in Likert scale design using the following criteria: Strongly Agree (1), Agree (2), Neutral (3), Disagree (4), Strongly Disagree (5). The third and final section of the survey contained two questions with an open-ended format to encourage student opinion and feedback.

### Data analysis

Analyses were performed using JMP, V9 statistical software (SAS Inst Inc, Cary, NC) on the 9 cultural competency questions of the survey with correlations completed with the demographic questions to evaluate response patterns. Descriptive statistics were generated for the various groups to address variations within and between health disciplines. Some of the data were analyzed using Spearman’s rank correlation statistic and the Kruskal-Wallis test. A significance level of P<.05 was selected to indicate statistical significance. Only student surveys with complete answers on rural upbringing and rural training questions were included. Survey responses were evaluated for the five health professional disciplines to determine perceived satisfaction with rural training and intent to seek work in rural locations. Likert scale responses were numbered to allow information to be identified by response groups. There was no weighting associated with the numerical assignments; all responses were equally considered.

A secondary data analyses was completed using SPSS 16 software program. Univariate analyses were used to compare the participant’s intent to choose a rural vs. an urban work setting. Univariate associations were made to determine the existence of any association between the participants’ preference for a rural or urban work setting and with the participants’ personal demographics. Chi-square analyses were used to contrast attitudes about rural training experiences and readiness for rural work settings. Relationships between demographic variables and willingness for interdisciplinary collaboration were assessed with a linear regression. Pearson r tests were used to assess the participants pride in rural background in relationship to their educational levels, graduate or undergraduate.

## Results

**Figure 1:**
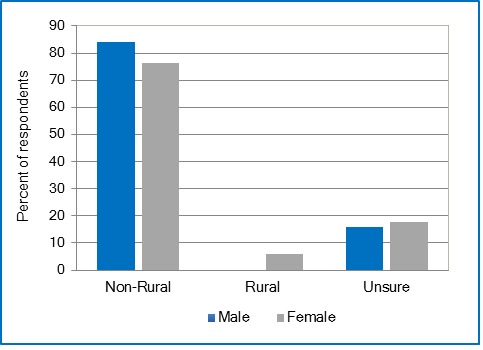
Relationship between gender and the intended size of city/town to practice

**Figure 2:**
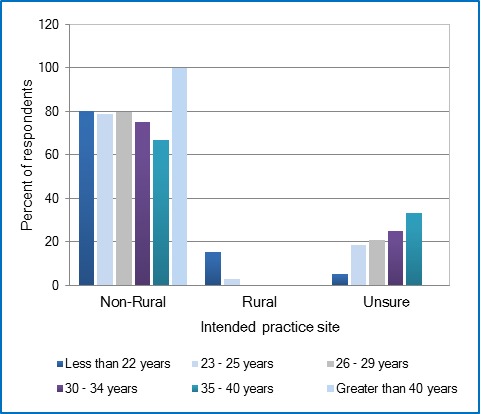
Relationship between age and the intended size of city/town to practice

One hundred-forty-three respondents out of a student population pool of 443 completed the survey yielding an excellent overall student population response rate of 32.3%. The individual school response rates were 16.6% (8 of 48) for Dentistry, 47.6% (10 or 21) for Dental Hygiene, 21.9% (39 of 178) for Nursing, 43.8% (49 of 112) for Pharmacy, and 43.8% (49 of 112) for Medicine. Despite lower response rates from Dentistry and Nursing, the sampling results are a valid reflection of the target student population’s opinions because the representative number of participants is still valid.Table 1 summarizes the demographic descriptors of the respondents. The majority of the 145 respondents were female (70%); 76% were between the ages of 23 to 29 years; and 87% were Caucasian. Most of the respondents in the older age groups (35 to 40 years and greater than 40 years) were from the Schools of Medicine and Nursing with only 5 of the total 143 respondents in this category. Twenty one percent (21%) of the respondents reported attending high school in areas designated as rural, with a population less than 2,500 residents. Only 12% of respondents reported going to high school in a large metropolitan area, with a population of 50,000 residents and more.Overall, 52% of the 145 participants intended to practice in a large metropolitan area with a population of 50,000 residents or more (Table 2). Only 4% of participants intended to practice in a designated rural area, all of which are females (n=6). Seventy per cent of respondents were unsure of what size setting they intended to practice in. Interestingly, only female participants intended to practice in a town with a population less than 10,000 residents (Figure 1). Females were significantly more likely to report intent to practice in a setting with a population of less than 50,000 among participants from the School of Medicine, using the Kruskal-Wallis test (p=.04). With regard to age as a predictive factor and N=145, none of the participants whose age was 26 years or greater intended to practice in a designated rural area (Figure 2). Participants 26 years and older comprised approximately one third of total respondents. Older students were significantly more likely to report intent to practice in a setting with a population of less than 50,000 among participants from the School of Pharmacy using Spearman’s Rank Correlation Coefficient (p=.04).

**Table 2:** West Virginia University (WVU) health professional students preferred practice site by size of town/city (N = 145)

Students preferred practice site by size of town/city	N	%
Size of Town/City		
Population > 100,000	37	26
Population 50,000 to 100,000	38	26
Population 20,000 to 49,999	21	14
Population 10,000 to 19,999	12	8
Population 2,500 to 9,999	6	4
Population < 2,500	6	4
Unsure	25	17

**Figure 3:**
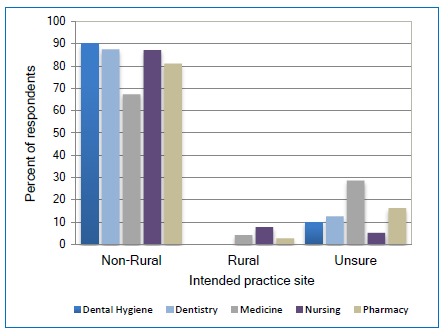
Relationship between discipline and the intended size of city/town to practice

While the percent of participants who reported their intent to practice in a designated rural area was low, the School of Nursing had the highest percentage at 8%, Medicine at 4% and Pharmacy at 3% (Figure 3 with N=145). However, there was a preference towards large metropolitan areas, with about half of the participants from the Schools of Medicine, Nursing and Pharmacy planning to practice in a location with a population of 50,000 or more residents.

**Figure 4:**
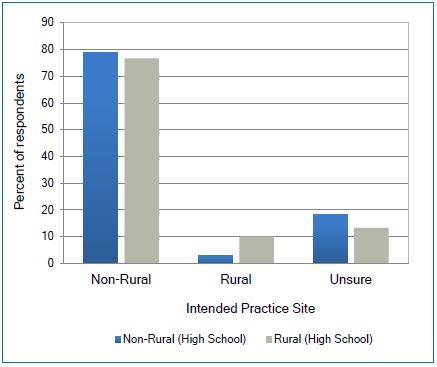
Relationship between size of city/town of high school and the intended size of city/town to practice

The majority of respondents who intended to practice in a designated rural area were those who attended high school in a designated rural area (Figure 4). However, this number also remained low, with only 10% of the total number participants who went to high school in a designated rural area reporting intent to practice in a rural area. Only 3% of the respondents who attended high school in a non-rural designated area intended to seek work in a rural area. Using Spearman’s Rank Correlation Coefficient there was a positive correlation among all disciplines between rural upbringing town size and the participants’ intent to practice in a town of the same size (p = .0011).

Those participants who intended to practice in a designated rural area of <2,500 were divided equally into two groups: those that had not completed a rural rotation and those that had completed a rural rotation.

**Figure 5:**
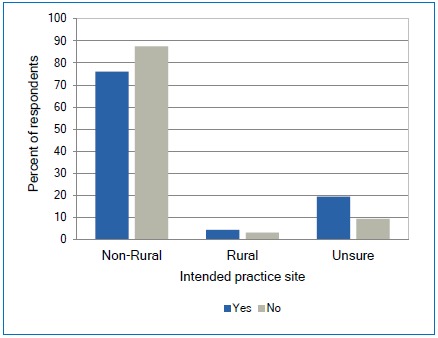
Relationship between completing a rural rotation and the intended size of city/town to practice

Interestingly, there was a trend that participants who had completed a rural rotation were more likely to be unsure of their intended practice site than those who had not completed a rural rotation, 19% vs. 9% respectively (Figure 5) with N =145.

**Figure 6:**
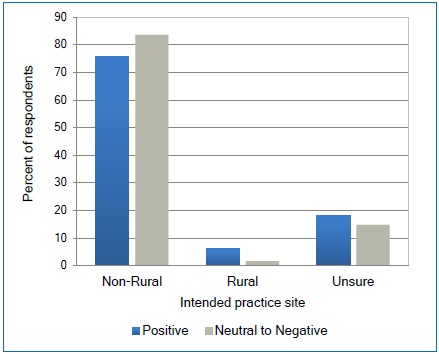
Relationship between attitude towards the rural rotation and the intended size of city/town to practice

Although the attitude results could not be validated during the first data analysis, it is interesting to note that there does not appear to be any difference in the attitude of respondents towards rural competency and their rural rotation when compared to intended practice site (Figure 6) with N =145. Not surprisingly, only 2% of respondents who reported a neutral to negative attitude towards rural competency and the rural rotation experience reported their intent to practice in a designated rural area. The percentage of respondents with a positive attitude towards rural competency and the rural rotation who intended to practice in a designated rural area was also low at 6%.

However, during the secondary data analyses, it was found that most, if not all, respondents had a positive attitude towards rural competency and the rural rotation experience. To explore satisfaction with rural rotation training experiences, a series of chi-square analyses were conducted. The entire group of participants endorsed their training about rural communities as effective (χ^2^_(1)_ = 105.800; p = <.0005). The entire group of participants felt their rural training had been satisfactory (χ^2^_(1)_ =116.485; p=<.0005). The multi-disciplinary group of participants rated themselves as competent regarding rural issues at a significant rate (χ^2^_(1)_ = 60.623; p = <.0005). On a related note, this same group of participants felt very confident about their rural training (χ^2^_(1)_ =127.259; p=<.0005). The group of students also felt their rural training had been adequate (χ^2^_(1)_ =116.485; p= <.0005).

Not surprisingly, respondents who intend to practice in a designated rural rotation had a slightly higher score in the attitude component of the survey, though the results cannot be completely validated from the results of this particular survey. Other personal factors which the authors theorize could be also related to willingness for rural practice included willingness to collaborate with other disciplines. Through the results revealed here (χ^2^_(1)_=117.279; p = <.0005) there seems to be a willingness for interdisciplinary collaboration. This sample also described themselves as proud to be from a rural area according to a positive correlational analyses (r=.344; p=<.0005; n=145). Pride in current rural residence were also positive correlations to rural upbringing (r =.174; p = <.0005; n = 145) and intent to practice in rural residence (r=.238; p=<.0005; n=145). This analysis shows that they are proud of their rural upbringing and rural residence, but their intent to practice is still requiring them to move into more populated areas.

## Discussion

As a health professional training institution serving a largely rural state, one important mission of the University is to provide students with the skills and education needed to practice effectively in a rural environment and improve the health of the underserved. Challenges involved in providing health to rural communities include recruitment and training about the unique characteristics of rural healthcare practice. Our study sought to learn about the background, rural training and practice location intentions of health professional students at an academic health sciences center in Appalachia. The participants in our survey acknowledged the importance of rural training and felt their training had a positive impact on their professional development.However, despite the emphasis on and perceived success of the rural health curriculum provided through this academic health sciences center, only a small percentage of respondents intended to practice in rural communities with a population less than 2,500 after graduation. The majority of students did not intend to practice in communities with populations smaller than even 50,000, but students who grew up in smaller towns were more likely to express intent to practice in areas with populations <50,000. The intent to practice in areas with populations >2,500 was consistent across all demographic groups, including those reporting rural high school attendance and completion of rural rotations. In fact, only 4% of the respondents reported the intent to practice in rural areas, designated as a population with less than 2,500 residents. All of the respondents reporting intent to work in rural settings were female and under 26 years of age. No respondents from either Dentistry or Dental Hygiene reported intent to practice in rural communities.Contrary to previous reports, the present findings suggest that rural upbringing is only a small factor on intent to practice in a rural setting among these multidisciplinary health professional students about to complete their training. Previous retrospective design studies suggested rural origin/upbringing as the strongest predictor for physicians and other medical professionals who were employed in rural areas.^2^^,^^6^^,^^10^^,^^12^ Some discrepancies between our findings and those of previous studies could be associated with the definition of rural used, participant differences and/or the retrospective methodology utilized. Our study utilized a far more conservative definition of rural which was a population size <2,500 based on the most stringent criteria for Census Urban Areas as compared to Daniels and colleagues use of the US Census Bureau’s 2000 dichotomous rural-urban variable (greater or less than 50,000).^10^^,^^17^ In addition, the present study queried prospective health sciences graduates about their future workplace location preferences rather than retrospectively reviewing their workplace locations years after graduation.Limitations of the present research project include the smaller sample size and recruitment of respondents from a single academic health sciences center, as well as the self-predictive response nature of the survey instrument. These results, however, are a valid reflection of the situation in West Virginia. Students also may have self-selected their participation by the project topic, thereby leading to the collecting of data from health professional students with more positive attitudes toward rural rotations and communities which may have affected external validity. Gender and racial-ethnic comparisons were difficult to conduct in this sample as there were far fewer respondents who were male or from racial ethnic minority backgrounds. There was also a difference in gender representation in the professions recruited for this project; for example, the disciplines of Nursing and Dental Hygiene had a far greater proportion of female students. Issues related to a rural community’s receptivity to female or racial minority health practitioners could influence one’s intent to seek rural health practice in a rural setting.^18^ All though there was a dominance of female gender in the sample for Nursing and Dental Hygiene, no sampling bias for the representation of these two professional areas is present for these professional areas seem to reflect the same conclusions as the other professions that were surveyed which were not predominantly female. Finally, the relationship between a student’s intent to practice and future job location and long-range retention is unclear. In the current job market, factors other than community preference may influence eventual practice location.Rural residents have substantial health care needs and their access to well-trained health professionals is often limited. One mission of WVRHEP and rural health sciences centers is to increase recruitment and retention of needed health care professionals to rural towns and isolated communities. Further study of health professional students’ pathways to rural practice is warranted. For example, a prospective longitudinal design tracking health professional students over the course of their training and careers is also likely to reveal significant developmental or work migration patterns. Gender, participants and/or discipline differences are also likely. Possible directions for further study within this setting could include whether the same intent to practice patterns exist among other disciplines at this university Health Sciences Center. Identification of effective incentives for students willing to seek positions in rural settings, and tracking the effectiveness of current rural rotation formats could be another direction. Also, other rural education experiences on willingness for rural practice using more stringent (pre-post or longitudinal) statistical designs could be used. Adjunctive supports such as discipline-specific or gender matched individual or group mentoring to discuss, plan and encourage new health professionals to seek rural health positions may also prove effective.On a broader level, comparisons of intent for rural practice between students in different health disciplines within the state and region would also provide important information. A suggestion would be plotting health professionals’ rural upbringing and intent to practice preferences with a census tract-based classification scheme. This approach considers not only the population size and density of a rural community but also relevant socio-demographic factors related to healthcare success. For example, the Rural Urban Continuum Codes which have a newer classification system that subdivides rural towns and communities into smaller subcategories. This could be utilized to more accurately characterize rural background as well as practice location choices.^19^ Findings from future investigations are also hoped to influence health policy and/or educational curriculums. This would improve the recruitment, training and retention of rural health professionals, as well as, provide needed advances in healthcare delivery for rural populations.

## Conclusions

In summary, the present results show that health professional students at this health sciences center generally do not intend to practice in a rural location. Only 4% of students expressed intent to work in rural communities, and all of these respondents were female and under 26 years of age. The intent to practice in a larger setting persisted regardless of discipline, gender, age, rural upbringing, completing a rural rotation, or having a positive attitude towards the rural rotation. With a more liberal definition of rural (population <50,000), there is a correlation between rural background and intent to practice in such areas; however, only 30% of the students in our sample reported an intent to practice in a town of this size, despite the majority (88%) coming from a community of this size.There are many possible theories as to why these students express intent to practice in non-rural settings. Perhaps the rural health rotations, despite an overall reported positive experience by these participants, give students greater insight into the challenges involved in rural health practice. It may be that, after completing their professional educations, students from rural areas seek different lifestyles than they experienced during adolescence. Another possibility is that there was a significant trend that participants who had completed a rural rotation were more likely to be unsure of their intended practice site than those that had not completed a rural rotation. Additionally, it could be that the rural rotation experience was a positive experience for the students and that they are now considering the rural setting as a possible practice site.Reimbursement and indebtedness may be significant factors in students’ ultimate decision of practice location. Contrary to previous work, our data did not find a correlation between rural upbringing or the completion of a rural rotation with intent to practice in a rural community. Our findings did confirm a general trend that students who attended high school in rural settings are more likely to seek work in smaller settings (populations <50,000). Unfortunately, though, not in the large numbers needed in rural West Virginia sites. More research is needed into the perceived disadvantages and deterrents to rural practice of health professions students. The healthcare needs for the rural underserved areas need to be adequately addressed making the solutions to the disadvantages and deterrents to health professions rural practice a continued priority.

### Conflict of Interest

The authors declare that they have no conflict of interest.
